# Hydrodilatation, corticosteroids and adhesive capsulitis: A randomized controlled trial

**DOI:** 10.1186/1471-2474-9-53

**Published:** 2008-04-19

**Authors:** Einar Kristian Tveitå, Rana Tariq, Sølve Sesseng, Niels Gunnar Juel, Erik Bautz-Holter

**Affiliations:** 1 Department of Physical Medicine and Rehabilitation, Ullevål University Hospital, Oslo, Norway; 2 Department of Radiology, Ullevål University Hospital, Oslo, Norway. University of Oslo, Norway

## Abstract

**Background:**

Hydrodilatation of the glenohumeral joint is by several authors reported to improve shoulder pain and range of motion for patients with adhesive capsulitis. Procedures described often involve the injection of corticosteroids, to which the reported treatment effects may be attributed. Any important contribution arising from the hydrodilatation procedure itself remains to be demonstrated.

**Methods:**

In this randomized trial, a hydrodilatation procedure including corticosteroids was compared with the injection of corticosteroids without dilatation. Patients were given three injections with two-week intervals, and all injections were given under fluoroscopic guidance. Outcome measures were the Shoulder Pain and Disability Index (SPADI) and measures of active and passive range of motion. Seventy-six patients were included and groups were compared six weeks after treatment. The study was designed as an open trial.

**Results:**

The groups showed a rather similar degree of improvement from baseline. According to a multiple regression analysis, the effect of dilatation was a mean improvement of 3 points (confidence interval: -5 to 11) on the SPADI 0–100 scale. T-tests did not demonstrate any significant between-group differences in range of motion.

**Conclusion:**

This study did not identify any important treatment effects resulting from three hydrodilatations that included steroid compared with three steroid injections alone.

**Trial registration:**

The study is registered in Current Controlled Trials with the registration number ISRCTN90567697.

## Background

Adhesive capsulitis is a common cause of shoulder pain and disability. It is characterized by a usually spontaneous onset of shoulder pain accompanied by progressive limitation of both active and passive glenohumeral movement. Both pain and stiffness tend to resolve spontaneously over months to years [1-4]. In 1945, Neviaser reported that the essential pathology of the condition "is a thickening and contraction of the capsule which becomes adherent to the humeral head" [5], thus causing reduced joint mobility in these patients. Some more recent arthroscopic investigations of frozen shoulder patients report no distinct intraarticular adhesions of this type [6-9], and the role of such adhesions is unclear. The finding of contractions of the joint capsule is supported though, as the studies describe a marked reduction in joint volume [6-9] due to contraction of collagenous tissue surrounding the joint [7].

During the decades following Neviaser's discovery, techniques were developed in order to loosen the reported contraction and adhesion. Distension arthrography (also known as hydrodilatation) is one of these techniques. It is in principle an injection into the glenohumeral joint under pressure. The hydrodilatation procedure was first described by Andrèn and Lundberg [10]. Since then, a number of investigators have studied the effects of hydrodilatation treatment, and several report beneficial results [11-21]. However, due to various weaknesses in study design, most of these studies are of limited value as evidence for a specific treatment effect from hydrodilatation. First and foremost, few of the studies claiming a treatment effect are randomized trials involving some sort of control group. Because these patients tend to recover spontaneously, a control group is essential in order to claim a treatment effect from the intervention. Secondly, treatment given in these studies was rarely restricted to mere hydrodilatation. Some interventions included a physiotherapy program or manipulation procedures. Most investigators used corticosteroids in addition to the dilatation procedure, and this may be important. Some studies investigating the effect of intraarticular steroids without dilatation have reported quicker improvement in pain in treated groups [22-24]. Rather similar treatment effects of oral corticosteroids have been found [25,26]. It is possible that the reported beneficial effects of the combined hydrodilatation/corticosteroid procedure rely on a corticosteroid effect, thus giving the dilatation part of the procedure unclear value.

No randomized trials have compared distension alone with placebo. The combined intervention of steroid injection and distension has been compared with steroid injection in three studies so far [15,27,28]. The study by Jacobs et al. [28] included a dilatation procedure injecting only a maximum of 10 ml, which is less than most other investigators have used. It is unclear whether this volume is sufficient to cause an effective distension of the joint capsule. In the two other studies, a volume of 20 ml was used in the distension groups. These studies were too small to conclude whether there was an additional treatment effect of the hydrodilatation procedure or not. The largest study had 45 patients available for analysis [13].

The aim of the present study is to investigate if the effect of dilatation represents an important contribution to overall treatment effects of a combined dilatation/steroid injection procedure.

## Methods

We conducted a randomized trial and compared the efficacy of hydrodilatation plus corticosteroid injection with the efficacy of corticosteroid injection without dilatation. In this way, we wanted to test the null hypothesis that neither of the interventions is superior to the other. The regional ethics committee granted ethical approval. The procedures followed protocol and complied with the Helsinki Declaration as revised in 1983 and current national ethical standards for such trials.

### Participants

Patients referred to the Ullevål University Hospital's Department of Physical medicine and Rehabilitation in the time period Dec. 1 2003 – June 1 2005 were considered for the study. Patients were assessed for eligibility according to the following criteria:

1. Limitation of passive movement in the glenohumeral joint compared with the unaffected side, more than 30 degrees for at least two of these three movements: forward flexion, abduction or external rotation. Patients with previous adhesive capsulitis in the opposite shoulder were accepted even if the differences between sides were somewhat smaller than 30 degrees. Patients were not eligible if they could not comply with range of motion measurement procedures due to e.g. excessive pain during measurements or huge difficulties in relaxing sufficiently to allow the investigator to make adequate recordings.

2. Pain in predominantly one shoulder lasting for more than 3 months, less than 2 years.

3. Willingness and ability to fill out shoulder self-report form.

The patients were included after informed consent unless they met any of the following criteria:

1. Unwillingness to participate in the trial.

2. Diabetes mellitus (DM). In our pilot study, we experienced problems for some DM patients in regulating blood sugar levels on the days following the injections. A study has shown that hydrodilatation may be less beneficial in diabetic patients [29]. All patients with DM were excluded from the study.

3. Trauma to the shoulder the last six months that required hospital care. We expected that patients with such a trauma generally would have a different prognosis and that this could cause problems when interpreting overall results.

4. Serious mental illness.

5. Age under 18 or over 70.

6. Various contraindications to injections: allergy to injection material, blood coagulation disorders.

7. Patients with cancer and patients not expected to be able to follow treatment or follow-up protocol for practical or other reasons.

8. Patients currently taking corticosteroid tablets.

9. Reduction of glenohumeral range of motion for reasons other than "classic" adhesive capsulitis, e.g. X-ray signs of glenohumeral arthritis, dislocation or full-thickness rotator cuff tears with displacement of the humeral head.

**Figure 1:**
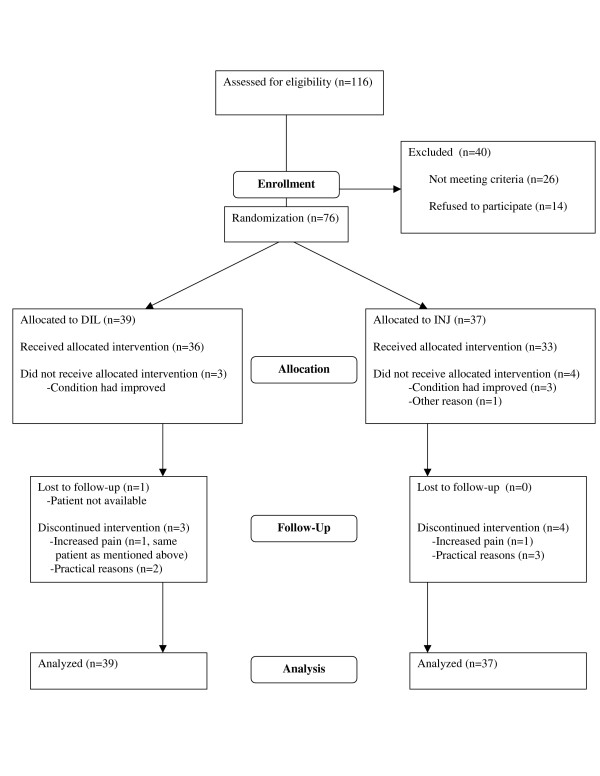
Patient flow.

Details of inclusion are given in Figure 1. 76 patients were included.

### Randomization

Randomization took place after baseline information had been gathered. Patients were randomized to one of the following interventions:

1. Injection (INJ) of a corticosteroid, a contrast agent and a local anesthetic.

2. Dilatation (DIL) with a corticosteroid, a contrast agent, local anesthetic plus saline necessary to obtain a dilatation and rupturing of the joint capsule.

Three injections with two-week intervals were planned for each patient. Randomization and allocation of patients was conducted on a patient-by-patient basis by an independent person (researcher) using the "minimization" method [30], with "loaded dice". Minimization was preferred to purely random allocation in order to reduce group differences at baseline. The chance of being allocated to a certain intervention group varied from 1/6 to 5/6 depending on initial SPADI [31] scores (stratifying for SPADI = 50 or SPADI > 50) and the number of patients already allocated to each intervention. 11 patients (30%) in the INJ group and 14 patients (36%) in the DIL group had baseline SPADI = 50. Patients were not informed of their actual assignments until the first injection was to be given. The randomization procedure was successful in the sense that it was followed according to protocol. Patient demographic and clinical characteristics at baseline are given in Table 1. Since we did not suspect any problems with the randomization, no baseline tests of imbalance were performed [32].

**Table 1:** Baseline demographic and clinical characteristics of included patients

**Variable**	**DIL group **(n = 39) Values are mean (SD) or n (n%) within group	**INJ group **(n = 37) Values are mean (SD) or n (n%) within group
**Age **(years)	52 (7)	51 (6)
**Female**	26 (67%)	19 (51%)
**Duration **(months)	7 (4)	7 (4)
**Left shoulder**	16 (41%)	20 (54%)
**Previous FS **(other shoulder)	8 (21%)	9 (24%)
**Patients with other shoulder problems previously**	12 (31%)	21 (57%)
**Patients who are smokers**	10 (26%)	13 (35%)
**Patients undergoing physiotherapy**	5 (13%)	8 (22%)
**Patients taking analgesics **(daily)	11 (28%)	9 (24%)
**Patients on sick leave**	15 (39%)	25 (68%)

### Interventions

The steps of the injection and dilatation procedures were documented with repeated X-ray examinations. The intervention procedures were as follows:

#### INJ

Arthrograms were performed according to the Kaye-Schneider technique [33]. The patients were placed supine on a table with an overhead X-ray tube and a supporting pillow under the opposite shoulder. Under image-intensified fluoroscopy a marker was placed over the glenohumeral joint space at about the junction of its middle and lower third. This point was then marked on the skin with a pen. The skin area was cleaned with an antiseptic. The joint was punctured by a needle (22 G intramuscular needle) and its position was checked frequently by fluoroscopy during the procedure. The needle was connected via a short tube to the syringe. An injection of 3–4 ml contrast medium (lopromide, Ultravist 300 Schering AG), 2 ml triamcinolone acetonide (Kenacort 10 mg/ml, Bristol-Myers Squibb) and 3–4 ml of local anesthetic (bupivacaine hydrochloride, Marcain 5 mg/ml, AstraZeneca) was given slowly, controlling visually that all fluid was injected into the joint.

#### DIL

When performing these injections, the syringe was filled with 4 ml of contrast medium, 2 ml triamcinolone acetonide, 4 ml local anesthetic and 10 ml saline. This injection of 20 ml in total was given to all patients undergoing the dilatation treatment. The fluid was injected very slowly into the joint. When resistance was met, injection was halted for a while, and then continued. During the injection, the joint was gradually distended, making especially the axillar and subscapular recesses more visible. The capsule would usually rupture in the wall of the subscapular recess, or sometimes in the wall of the bicipital or axillary recesses. This was recorded as a loss of resistance and contrast leakage was identified by fluoroscopy. If rupture had not occurred, more Ultravist/Marcain was injected until rupture. The needle was then ejected. Rupture occurred in all patients but one belonging to the dilatation group.

Rupture also occurred in four of the patients belonging to the injection group, even though the injection volume was kept as low as possible to prevent this. The volume given was judged to be necessary to inject corticosteroid, local anesthetic and contrast material sufficient to confirm the injection site. Mean volumes injected in the INJ group on the three occasions were 8 ml (SD:4), 7 ml (SD:2) and 7 ml (SD:2). The overall mean in this group was 7 ml (SD:3). Mean volumes in the DIL group were 21 ml (SD:3), 20 ml (SD:1) and 21 ml (SD:2). Overall mean was 21 ml (SD:2). The number of patients in the DIL group who received more than 20 ml were 8, 3 and 4 on each of the three occasions.

At baseline, some patients were taking oral medication for shoulder pain, usually acetaminophen or various NSAIDs. A few were receiving physiotherapy to improve their shoulder condition. Patients were not instructed to attend to any specific physiotherapy program or manipulation procedure, but were allowed to proceed with their current treatment program if they wished. No patients were prescribed new physiotherapy programs during the study. Pain medication, physiotherapy and sick leave during the study were organized by the patients' primary care clinicians who were not informed of the patients' treatment allocation.

### Follow-up and assessment of outcome

The following parameters were chosen as outcome measures and registered at baseline (inclusion) and at follow-up, 6 weeks after the last injection:

1. SPADI score. SPADI is a self-administered instrument that measures pain and disability associated with shoulder disease [31]. It consists of five pain and eight disability items each measured on a visual analogue scale. Pain and disability subscales are calculated as the mean of the corresponding items on a 0–100 scale, the highest score indicating the most severe pain and disability. The total score is calculated as the average of the pain and disability subscales. The score used in our study was a Norwegian version [34], translated according to internationally accepted guidelines [35]. SPADI has been used in previous randomized trials investigating treatment effects in frozen shoulder populations [11,23,26]. Psychometric properties of the index have been tested in shoulder patients and indicate acceptable validity for group comparisons [36-38].

2. Active and passive range of motion (AROM and PROM). Measurements of range of motion (ROM) were made in four different directions of movement: forward flexion and abduction from neutral (patient standing) and external/internal rotation from a position of approximately 45° of abduction (patient in supine position). PROM measurements were performed with scapula manually stabilized and end-point was determined at the point where resistance to the movement was felt and before the scapula began to move (aiming to measure *glenohumeral *ROM). For active movements, the patient was instructed to move the arm as far as possible in the requested direction with minimal additional movement. Scapular movement was not restricted, meaning that *total shoulder *ROM in each direction was measured in this case. The reliability of ROM measurements was estimated (separate study) and ICC ranged from 0.61 to 0.93 for the various movements. A Cybex Electronic Digital Inclinometer (EDI 320, Cybex Inc., Ronkonkoma, NY) was used.

No specific treatment was planned between the injections or between the last injection and follow-up. All data was recorded by Nov. 1 2005. Follow-up measurements were made by the same independent person, who was experienced in range of motion measurements. The study was an open trial where neither participants nor outcome assessors were blinded.

### Statistical analyses

Analyses were conducted according to the intention-to-treat (ITT) principle [39]. Linear regression analysis [40] was used to compare SPADI score improvement in the two treatment groups. This type of analysis was chosen according to recommendations for controlling for baseline differences when reporting results from randomized trials [41,42]. According to the recommendations by Roberts and Torgerson [32] and Senn [41], baseline variables of potential prognostic value were identified beforehand and then fitted in the analysis. Literature and pilot studies were used to select variables. These were "treatment assignment", "gender", "age", "baseline SPADI", "sick leave at baseline", "baseline pain medication", "duration of condition" and "previous shoulder problems". All variables except "age" and "baseline SPADI" were dichotomized. Comparisons for ROM were performed by independent t-tests.

### Minimal clinical difference and sample size

A pilot study and previous studies were used to plan the size of our study. We expected a between-patient standard deviation (for ΔSPADI) of approximately 17 points. In a previous study [23], a difference in SPADI score of 10 points or more was chosen as a minimal clinically significant difference in shoulder pain and function. Published data on this point is rather limited, but for practical reasons, we adopted the same figure in our sample size calculations. With an alpha (p-value) of 5% and a power of 80%, this would result in a necessary sample size of 44 patients per group for a two-sided t-test. We judged that a somewhat smaller sample size would be sufficient for our purpose because we expected the regression analysis to have greater statistical power to detect a treatment effect than a usual t-test [42]. No interim analyses were planned or conducted. All statistical analyses were carried out by using the software package SPSS 13.0 for Windows^® ^(SPSS, Chicago, IL, USA) and the recommendations by Andy Field [43].

## Results

### Patient flow

The flow of participants through each stage of the trial is reported by a CONSORT [44] flow diagram (Figure 1). There was an unexpected restriction in treatment capacity, which caused a waiting period for most patients before any injections were given. Mean time from inclusion (baseline assessment) to the first injection was received was 8.2 weeks in the INJ group and 8.6 weeks in the DIL group. 76 patients were included, of which 62 patients received treatment according to protocol. The main reason for lack of compliance was improvement of the condition before any treatment had been given (n = 6), or patients were not able to come to all three appointments (n = 5). Mean baseline SPADI score for the patients who were not given any injections because they felt their condition had improved, was 53 (SD 18), and mean improvement for these patients was 40 (SD 20). Follow-up data for patients that were not given all injections were collected at the same time point as if all planned injections had been given. One patient moved to another part of the country and was not available for follow-up. This patient belonged to the DIL group and had a baseline SPADI score of 40, which was adopted as the follow-up score for this patient. For the 75 other patients, original follow-up scores were used in the computations. One patient who belonged to the INJ group was given 3 corticosteroid injections by a different doctor before any of the planned injections had been given. Our staff did not administer any injections to this patient, who had a baseline SPADI score of 93 and a follow-up score of 74.

### Treatment effects

Both groups had significant improvement from baseline, as could be expected for patients with this condition. When baseline variables are adjusted for, the results indicate a mean difference in ΔSPADI between treatment groups of 3 points (Table 2). The observed difference is in favor of the dilatation group, but is not significant (CI: -5 to 11). High age or high baseline SPADI score indicate large improvement in SPADI during treatment, according to the simple regression analysis when variables are investigated one at a time. "Baseline SPADI score" and "Baseline pain medication" appear as significant predictors when all variables are analyzed simultaneously. The full model explains 53% of the total variance in ΔSPADI. Residuals are normally distributed and there is no evidence of heteroscedasticity [43].

**Table 2:** Results of the regression analysis; dependent variable "SPADI improvement"

**Variable**	**Simple regression** (variables analyzed separately)		**Multiple regression** (full model)	
	Coefficients	95% CI	Coefficients	95% CI

**Treatment** (INJ coded 0, DIL coded 1)	1	(-9 to 11)	3	(-5 to 11)
**Gender** (female coded 0, male coded 1)	-1	(-11 to 9)	3	(-5 to 11)
**Age** (years)	**0.9**	**(0.1 to 1.7)**	0.4	(-0.2 to 1.0)
**Baseline SPADI score**	**0.7**	**(0.5 to 0.9)**	**0.9**	**(0.7 to 1.1)**
**Baseline pain medication** (< 1 tablet/day coded 0, 1 tablet/day or more coded 1)	3	(-8 to 14)	**-14**	**(-23 to -4)**
**Sick leave at baseline** ("No" coded 0, "Yes" coded 1)	-1	(-11 to 9)	-5	(-14 to 3)
**Previous shoulder problems** (other than capsulitis) ("No" coded 0, "Yes" coded 1)	-3	(-13 to 7)	-4	(-12 to 4)
**Duration of condition at baseline** (< 6 months coded 0, 6 months or more coded 1)	-8	(-18 to 2)	0	(-8 to 7)

Details regarding mean (SD) values for SPADI and ROM for baseline, change and follow-up scores are given in Table 3. According to independent t-test comparisons there are no statistically significant group differences for any of the ROM measures.

**Table 3:** Results for SPADI and ROM

**Movement**	**Baseline** Mean (SD)		**Change** Mean (SD) or Mean (95% CI)			**Follow-up** Mean (SD) or Mean (95% CI)		
	**DIL**	**INJ**	**DIL**	**INJ**	**Group diff**.	**DIL**	**INJ**	**Group diff**.

**SPADI**	59 (20)	63 (20)	*-39 (21)*	*-38 (22)*	*-1 (-11 to 9)*	20 (17)	26 (19)	-6 (-14 to 2)
**Passive abduction**	31 (11)	31 (11)	*14 (12)*	*14 (10)*	*-1 (-6 to 4)*	44 (12)	46 (13)	-2 (-8 to 4)
**Passive forward flexion**	46 (17)	48 (14)	*15 (18)*	*16 (13)*	*-1 (-8 to 6)*	61 (13)	65 (12)	-3 (-9 to 2)
**Passive ext. rotation**	16 (14)	19 (13)	*11 (14)*	*10 (11)*	*1 (-5 to 6)*	27 (17)	29 (16)	-2 (-10 to 5)
**Passive int. rotation**	32 (13)	34 (14)	*13 (10)*	*15 (12)*	*-2 (-7 to 3)*	45 (12)	48 (15)	-3 (-9 to 4)
**Active abduction**	55 (20)	57 (21)	*31 (30)*	*26 (33)*	*5 (-10 to 19)*	86 (34)	83 (37)	4 (-13 to 20)
**Active forward flexion**	89 (25)	87 (24)	*28 (31)*	*29 (28)*	*-1 (-15 to 12)*	117 (28)	116 (30)	1 (-12 to 15)
**Active ext. rotation**	22 (16)	23 (15)	*18 (16)*	*14 (12)*	*3 (-3 to 10)*	39 (20)	37 (17)	3 (-6 to 11)
**Active int. rotation**	45 (16)	46 (15)	*22 (16)*	*21 (15)*	*2 (-5 to 9)*	68 (17)	66 (18)	1 (-7 to 9)

At follow-up, five patients (13%) in the DIL group and three patients (8%) in the INJ group were taking analgesics on a daily basis. Eleven patients (28%) in the DIL group and twenty-three patients (62%) in the INJ group were on sick leave.

### Side effects and adverse reactions

Patients recorded pain intensity related to the injection procedures. Most patients reported "no pain" or "discomfort" when describing the procedure. However, six patients in the INJ group and five patients in the DIL group felt that the injections were very painful. Other possible side effects were reported by 20 patients in the INJ group and 14 patients in the DIL group. These were usually mild and lasted only for a few days. Most frequent were complaints over flushing or disturbances in heat regulation (INJ group n = 13, DIL group n = 9). Two patients in each group reported a minor loss of sensation and motor control in their affected arm. Some patients complained over loss of sleep, nausea or dizziness. One patient in the DIL group developed a glenohumeral joint infection which was identified 5 days after the last injection. He immediately underwent arthroscopic surgery and was treated with infusions of cloxacillin for two weeks, with a good result. His baseline SPADI score was 38, and the score at follow-up was 50. One patient in the INJ group developed breast cancer during the study period. Formal statistical tests to compare adverse reactions in the two groups were not made.

## Discussion

Our study has not identified significant between-group differences in main outcomes after treatment of the two intervention groups. There may be several reasons for this result. One may be that the hydrodilatation procedure itself is of little or no additional value in improving patient outcome. However, we cannot conclude that this is the case, even though mean improvement was almost similar in the two groups. Confidence intervals do not exclude a difference in SPADI improvement larger than the 10-point difference chosen beforehand to be the minimal clinically significant difference, meaning that an important treatment effect from dilatation cannot be excluded.

The procedure of hydrodilatation is thought to exert its positive effects by improving glenohumeral mobility via stretching or rupturing of the joint capsule. Gam et al. [15] reported significant improvement in various ROM measures in the group treated with distension plus steroid compared with the group treated with steroid alone. In the present study, the measures of range of motion were almost equal in the two groups at follow-up, a result much in line with the findings of Corbeil et al. [27]. The dilatation procedure may not be able to "release" the contracted ligaments that cause restricted ROM. It is possible, or perhaps even likely, that other structures than the walls of the subscapular recess must rupture for such effects to occur.

The observed lack of difference between the two groups may stem from an inadequate procedure of dilatation. We used a total volume ranging from 20 to 30 ml, the actual volume given depending on joint capsule distensibility before rupture. Other investigators have used larger amounts of fluid, e.g. Buchbinder et al. [11] who used a mean of 43 ml in the distension group. Our choice of procedure was based on the notion that continuing the injection when a rupture had occurred would give little additional distension of the capsule, but instead cause extra-articular deposition of injected material. Injection volume in the INJ group was limited to 8–10 ml, but in some cases rupture occurred even with this small volume. There may have been a "dilatation" effect in some of the patients in the INJ group, making it more difficult to identify possible group differences.

Several investigators recommending the hydrodilatation procedure base these recommendations on results of studies where patients have been presented with a standardized protocol of physiotherapy or manipulation [12-14,16,19-21]. In our study, very few patients were undergoing physiotherapy during the trial. It is unclear whether the specific effect of hydrodilatation depends on a combination with these interventions.

In most cases, adhesive capsulitis is a temporary condition. This makes it crucial to consider the time factor when interpreting treatment results. In this study, patients with shoulder pain for less than three months were not included because we were afraid it would be difficult to distinguish between typical bursitis and developing capsulitis in this early phase. It may be that dilatation can occur more easily in the early stages of the condition, and the intervention may be of special value to these patients. We do not know what the results would have been if patients with acute frozen shoulder were also included in the study.

A weakness of our study was an unexpected restriction in treatment capacity, which caused a waiting period for most patients before any injections were given. Baseline assessments were made at inclusion, and mean time from inclusion to treatment was about two months. Some patients experienced spontaneous recovery in this period, leaving a smaller margin for improvement as a result of treatment. Also the waiting period may have reduced the effect of dilatation due to more rigid structures in or around the joint capsule.

The waiting period may also have led to the larger variation in patient outcome (ΔSPADI) than we had expected (SDs were 21–22 compared to 17). On the other hand, the regression model appears to explain a large proportion (53%) of the variance in ΔSPADI, and it is our view that the trial is not essentially undersized for this type of study.

Patients were not blinded, and this may have introduced bias, particularly since the primary outcome is a self-reported form. Patients were told that they would all receive corticosteroid injections, and some of them would receive large volume injections in order to distend contractions in tissues surrounding the joint. Information was kept as balanced as possible, but some patients probably had the impression that hydrodilatation was the more "sophisticated" approach. This may have biased results in favor of this procedure.

The number of injections used in our study was based on pilot studies and previous studies. The majority of researchers have given only one injection. Some use three injections, while Gam and colleagues [15] report giving up to six injections. Although grossly underpowered, Gam's study suggests that dilatation may be superior to simple injection in improving ROM. Our choice was to use a number that was closer to the "usual" approach, yet was not essentially inferior to Gam's regimen. However, using three injections instead of one may have been unfortunate given the aim of the study, which was to identify treatment effects from hydrodilatation. It is likely that a steroid injection may improve this condition. Repeating injections may add to this, leaving a smaller margin for improvement. Whether hydrodilatation effects cumulate if injections are repeated is more unclear. It is possible that the specific effects of hydrodilatation might have been easier to identify if only one injection had been given.

We have chosen to compare treatment effects by using the method of a randomized controlled trial. Given the random allocation of participants, we allow for the possibility that the treatment groups have important differences in various respects. Using a regression model, we have tried to control for variables that we expected to be relevant. However, there may have been important group differences at baseline that were not revealed or controlled for.

## Conclusion

In this study, we investigated treatment effects in patients with adhesive capsulitis who received intraarticular injections either with or without a dilatation procedure. No significant differences for selected outcomes between the two treatment groups were recorded, but it is possible that hydrodilatation may have an important treatment effect in some cases. As for other interventions for shoulder disorders, there is a need for future trials investigating the effect of shoulder injections in patients with adhesive capsulitis.

## Competing interests

The author(s) declares that they have no competing interests.

## Authors' contributions

All authors contributed to study design. EKT recruited the patients, performed the statistical analysis and drafted the manuscript. RT and SS performed the injections. NGJ and EBH helped to draft the manuscript. All authors read and approved the final manuscript.

## Pre-publication history

The pre-publication history for this paper can be accessed here:



## References

[B1] Binder AI, Bulgen DY, Hazleman BL, Roberts S (1984). Frozen shoulder. A long-term prospective study. Ann Rheum Dis.

[B2] Grey RG (1978). The natural history of "idiopathic" frozen shoulder. J Bone Joint Surg Am.

[B3] Reeves B (1975). The natural history of the frozen shoulder syndrome. Scand J Rheumatol.

[B4] Shaffer B, Tibone JE, Kerlan RK (1992). Frozen shoulder. A long-term follow-up. J Bone Joint Surg Am.

[B5] Neviaser JS (1945). Adhesive capsulitis of the shoulder. J Bone Joint Surg.

[B6] Ha'eri GB, Maitland A (1981). Arthroscopic findings in the frozen shoulder. J Rheumatol.

[B7] Kilian O, Kriegsmann J, Berghauser K, Stahl JP, Horas U, Heerdegen R (2001). Die "frozen shoulder". Arthroskopische, histologische und elektronmikroskopische Untersuchungen. Chirurg.

[B8] Uitvlugt G, Detrisac DA, Johnson LL, Austin MD, Johnson C (1993). Arthroscopic observations before and after manipulation of frozen shoulder. Arthroscopy.

[B9] Wiley AM (1991). Arthroscopic appearance of frozen shoulder. Arthroscopy.

[B10] Andren L, Lundberg BJ (1965). Treatment of rigid shoulder by joint distension during arthrography. Acta Orthop Scand.

[B11] Buchbinder R, S Green, A Forbes, S Hall, G Lawler (2004). Arthrographic joint distension with saline and steroid improves function and reduces pain in patients with painful stiff shoulder: results of a randomised, double blind, placebo controlled trial. Ann Rheum Dis.

[B12] Callinan N, McPherson S, Cleaveland S, Voss DG, Rainville D, Tokar N (2003). Effectiveness of hydroplasty and therapeutic exercise for treatment of frozen shoulder. J Hand Ther.

[B13] Ekelund AL, Rydell N (1992). Combination treatment for adhesive capsulitis of the shoulder. Clin Orthop Relat Res.

[B14] Fareed DO, Gallivan WR (1989). Office management of frozen shoulder syndrome. Treatment with hydraulic distension under local anesthesia. Clin Orthop Relat Res.

[B15] Gam AN, Schydlowsky P, Rossel I, Remvik L, Jensen EM (1998). Treatment of "frozen shoulder" with distention and glucocorticoid compared with glucocorticoid alone. Scand J Rheumatol.

[B16] Hsu SY, Chan KM (1991). Arthroscopic distension in the management of frozen shoulder. Int Orthop.

[B17] Morency G, Dussault RG, Robillard P, Samson L (1989). Arthrographie distensive dans le traitement de la capsulite adhésive de l'épaule. Can Assoc Radiol J.

[B18] Mulcahy KA, Baxter AD, Oni OO, Finlay D (1994). The value of shoulder distension arthrography with intraarticular injection of steroid and local anaesthetic: a follow-up study. Br J Radiol.

[B19] Sharma RK, Bajekal RA, Bhan S (1993). Frozen shoulder syndrome. A comparison of hydraulic distension and manipulation. Int Orthop.

[B20] van Royen BJ, Pavlov PW (1996). Treatment of frozen shoulder by distension and manipulation under local anaesthesia. Int Orthop.

[B21] Wybier M, Parlier-Cuau C, Baque MC, Champsaur P, Haddad A, Laredo JD (1997). Distension arthrography in frozen shoulder syndrome. Semin Musculoskelet Radiol.

[B22] Bulgen DY, Binder AI, Hazleman BL, Dutton J, Roberts S (1984). Frozen shoulder. Prospective clinical study with an evaluation of three treatment regimens. Ann Rheum Dis.

[B23] Carette S, Moffet H, Tardif J, Bessette L, Morin F, Frémont P, Bykerk V, Thorne C, Bell M, Bensen W, Blanchette C (2003). Intraarticular corticosteroids, supervised physiotherapy, or a combination of the two in the treatment of adhesive capsulitis of the shoulder: A placebo-controlled trial. Arthritis & Rheumatism.

[B24] Ryans I, Montgomery A, Galway R, Kernohan WG, McKane R (2005). A randomized controlled trial of intra-articular triamcinolone and/or physiotherapy in shoulder capsulitis. Rheumatology.

[B25] Binder A, Hazleman BL, Parr G, Roberts S (1986). A controlled study of oral prednisolone in frozen shoulder. Br J Rheumatol.

[B26] Buchbinder R, Hoving JL, Green S, Hall S, Forbes A, Nash P (2004). Short course prednisolone for adhesive capsulitis (frozen shoulder or stiff painful shoulder): a randomised, double blind, placebo controlled trial. Ann Rheum Dis.

[B27] Corbeil V, Dussault RG, Leduc BE, Fleury J (1992). Capsulite rétractile de l'épaule: étude comparative de l'arthrographie avec corticothérapie intra-articulaire avec ou sans distension capsulaire. Can Assoc Radiol J.

[B28] Jacobs LG, Barton MA, Wallace WA, Ferrousis J, Dunn NA, Bossingham DH (1991). Intraarticular distension and steroids in the management of capsulitis of the shoulder. BMJ.

[B29] Bell S, Coghlan J, Richardson M (2003). Hydrodilatation in the management of shoulder capsulitis. Australas Radiol.

[B30] Treasure T, MacRae KD (1998). Minimisation: the platinum standard for trials?. BMJ.

[B31] Roach KE, Budiman-Mak E, Songsiridej N, Lertratanakul (1991). Development of a shoulder pain and disability index. Arthritis Care Res.

[B32] Roberts C, Torgerson DJ (1999). Baseline imbalance in randomised controlled trials. BMJ.

[B33] Schneider R, Ghelman B, Kaye JJ (1975). A simplified injection technique for shoulder arthrography. Radiology.

[B34] Juel NG (2007). Norsk fysikalsk medisin.

[B35] Beaton DE, Bombardier C, Guillemin F, Ferraz MB (2000). Guidelines for the process of cross-cultural adaptation of self-report measures. SPINE.

[B36] Beaton DE, Richards RR (1996). Measuring function of the shoulder: a cross-sectional comparison of five questionnaires. J Bone Joint Surg Am.

[B37] Beaton DE, Richards RR (1998). Assessing the reliability and responsiveness of 5 shoulder questionnaires. J Shoulder Elbow Surg.

[B38] Heald SL, Riddle DL, Lamb RL (1997). The Shoulder Pain and Disability Index: the construct validity and responsiveness of a region-specific disability measure. Phys Ther.

[B39] Lewis JA, Machin D (1983). Intention to treat – who should use ITT?. Brit J Cancer.

[B40] Altman DG (1991). Practical statistics for medical research.

[B41] Senn S (1991). Baseline comparisons in randomized clinical trials. Stat Med.

[B42] Vickers AJ, Altman DG (2001). Analysing controlled trials with baseline and follow up measurements. BMJ.

[B43] Field A (2005). Discovering statistics using SPSS.

[B44] Moher D, Schulz KF, Altman DG (2001). The CONSORT Statement: Revised recommendations for improving the quality of reports of group randomized trials. JAMA.

